# 400 nm femtosecond laser irradiation eradicates mature MRSA biofilms and inhibits their formation via quorum sensing dysregulation

**DOI:** 10.1038/s41598-026-64350-w

**Published:** 2026-08-12

**Authors:** Esraa Ahmed, Ahmed O. El-Gendy, Michael R. Hamblin, Tarek Mohamed

**Affiliations:** 1 Laser Institute for Research and Applications LIRA, Beni-Suef University, Beni-Suef, 62511 Egypt; 2 Faculty of Pharmacy, Department of Microbiology and Immunology, Beni-Suef University, Beni-Suef, 62514 Egypt; 3 Laser Research Centre, Faculty of Health Science, University of Johannesburg, Doornfontein, 2028 South Africa

**Keywords:** Femtosecond laser, Antibacterial photodynamic therapy, Quorum-sensing, MRSA, Decoupling gene expression, *Ica* operon, Biofilm biomass, Biotechnology, Microbiology

## Abstract

Biofilm infections caused by methicillin-resistant *Staphylococcus aureus* (MRSA) represent a critical global health challenge. This study comprehensively assessed the anti-biofilm efficacy of femtosecond laser treatment for both the prevention of biofilm formation and the destruction of pre-formed mature biofilms. The experimental setup utilized the INSPIRE HF100 laser system, which was pumped by the MAI TAI HP laser system, to provide femtosecond laser pulses at a wavelength of 400 nm, varying both the exposure duration and average power. We found that all the femtosecond laser treatments effectively impaired MRSA’s ability to form biofilms, with the most potent effect observed at an average power of 50 mW for 45 min, with a significant 75.3% reduction in biofilm viability, 44.5% biomass reduction, and 60–85% reduction in metabolic activity achieved at 45 min (*p* < 0.0001 by ANOVA and Tukey test). Furthermore, the destruction of pre-formed biofilms was strongly dependent on the exposure duration, with 69% reduction in biofilm viability, 27.7% biomass reduction, and a 55.7% reduction in metabolic activity achieved at 45 min (*p* < 0.0001 by ANOVA and Tukey test). Gene expression analysis of 14 selected genes supports a model in which the femtosecond laser induced a significant transcriptional dysregulation across the quorum-sensing network, suggestive of a broad-scale attenuation of regulatory activity and leading to a functional failure in biofilm establishment, despite the transcriptional upregulation of adhesion genes. The integration of our quantitative (CFU), physical (CV), metabolic (MTT), morphological (SEM), and molecular (RT-qPCR) data provides a comprehensive “multi-dimensional” understanding of how 400 nm femtosecond laser irradiation mitigates MRSA biofilm formation and facilitates the eradication of mature biofilms. This work reports the optimization of femtosecond laser parameters for both anti-virulence and biofilm destruction, positioning it as a promising antibacterial modality against multidrug-resistant bacterial infections.

**Supplementary Information:**

The online version contains supplementary material available at 10.1038/s41598-026-64350-w.

## Introduction

The growing incidence of chronic multidrug-resistant (MDR) bacterial infections poses a global healthcare and economic threat^1,2,3^, especially bacterial infections caused by persistent bacterial biofilms, which exhibit a significantly enhanced resistance (up to 1000-fold) to antibiotics, and immune evasion capability^4,5,6^. Foremost among these bacteria is methicillin-resistant *Staphylococcus aureus* (MRSA), an opportunistic pathogen colonizing over 30% of the global population^7^ and responsible for both localized and systemic, potentially life-threatening infections^8^.

The progression, severity, and pathogenicity of bacterial infections are primarily governed by a variety of bacterial products known as “virulence factors,” which may be cell-surface associated or secreted following infection, often displaying strain-specific diversity and functional heterogeneity^9^. The ability of a bacterial pathogen to form biofilms is regarded as a major virulence factor^10^. A biofilm is a heterogeneous 3-dimensional growth state where microbial communities are wrapped in a self-produced protective extracellular polymeric substances (EPS) matrix containing necrotic tissue^11^. Biofilms are highly adaptive, resistant bacterial growth states designed to survive in harsh environmental conditions, because they act as a barrier to both antibiotics and the host immune system^6^. Unlike bacteria in a planktonic state, it is difficult to treat and eradicate biofilm infections^12,13^. Clinically, biofilms cause approximately 65–80% of infections^14,15^, where they often lead to severe illness with a prolonged hospital stay, increased costs, and high mortality^16^.

The expression of bacterial virulence factors can be influenced by genetic and environmental conditions. Bacterial metabolic pathways modulate the pathogens’ virulence, resulting in phenotypic alterations^17^. These alterations are connected to the degree of expression of genes encoding these factors, and bacteria precisely regulate this expression to avoid energy waste^18^. The mechanisms underlying this regulation involve chemical cell-to-cell signaling (autoinducers) between bacterial cells, as well as between bacteria and the host, forming a complex regulatory network known as the quorum-sensing system^19^. Quorum sensing enables bacteria to modulate gene expression and control virulence factors (such as motility, adhesion, biofilm formation, invasion, and immune evasion) in response to their population density, consequently playing a significant role in bacterial infection^20,21^. Understanding and targeting these virulence factors, as well as their functions, offers promising prospects to prevent and combat challenging bacterial biofilm infections^22,23^.

The multifactorial nature of biofilms combined with the emergence of multidrug-resistant pathogens represents a challenge to existing antibacterial therapeutic approaches, which sometimes have a narrow spectrum of activity, rendering them ineffective^24^. In contrast, different novel antivirulence and quorum quenching drugs have been investigated to inhibit bacterial adhesion and invasion of host tissue, to disrupt the quorum-sensing system, interfere with biofilm formation, prevent toxin secretion, and modulate virulence gene expression. Despite their potential, most of these are still in the early stages of development, and clinical trials have yet to be conducted^18,19^. These drawbacks underscore the need for a robust alternative broad-spectrum strategy to effectively target persistent biofilm infections^25^.

Our increasing understanding of microbial biofilms has fundamentally reshaped the strategies employed against these challenging infections. One highly promising treatment strategy is laser-based antimicrobial photodynamic therapy (aPDT), a rapid and non-invasive approach with potential for eradicating targeted bacterial pathogens^21,26^. Owing to its multi-mechanistic mode of action, aPDT has only a minimal risk for the development of bacterial resistance^27^. Unfortunately, one of the major barriers is the limited number of studies addressing the use of aPDT as an antivirulence approach. The precision and targeted delivery of laser irradiation augments its therapeutic efficacy, allowing optimal optical tissue penetration, an effective exposure duration, and adequate fluence without harming adjacent tissue.

This report describes a potential approach for mitigating MRSA biofilm infections using 400 nm femtosecond laser-based aPDT. Our objectives were to: (1) evaluate the potential antivirulence efficacy of various femtosecond laser treatments in attenuating the biofilm formation capability; (2) uncover the transcriptional and regulatory network changes associated with femtosecond laser-based aPDT; (3) comparatively assess and optimize the femtosecond laser-based aPDT parameters for maximum biofilm destruction. To the best of our knowledge, this is the first study to evaluate the effectiveness of sublethal doses of femtosecond laser-based aPDT as an antivirulence strategy targeting biofilm formation and destruction, aiming to optimize treatment parameters in anticipation of future clinical testing.

## Materials and methods

### Microorganisms and culture conditions

In this report, methicillin-resistant *Staphylococcus aureus* (MRSA) ATCC 43300 was retrieved from glycerol stocks, cultured in Brain Heart Infusion (BHI) broth, and incubated overnight (14–16 h) with shaking under aerobic conditions at 37 °C. All experimental procedures were conducted under sterile conditions within a Class II biological safety cabinet (MSC-Advantage™).

### Femtosecond laser systems and exposure setup

**Fig. 1:**
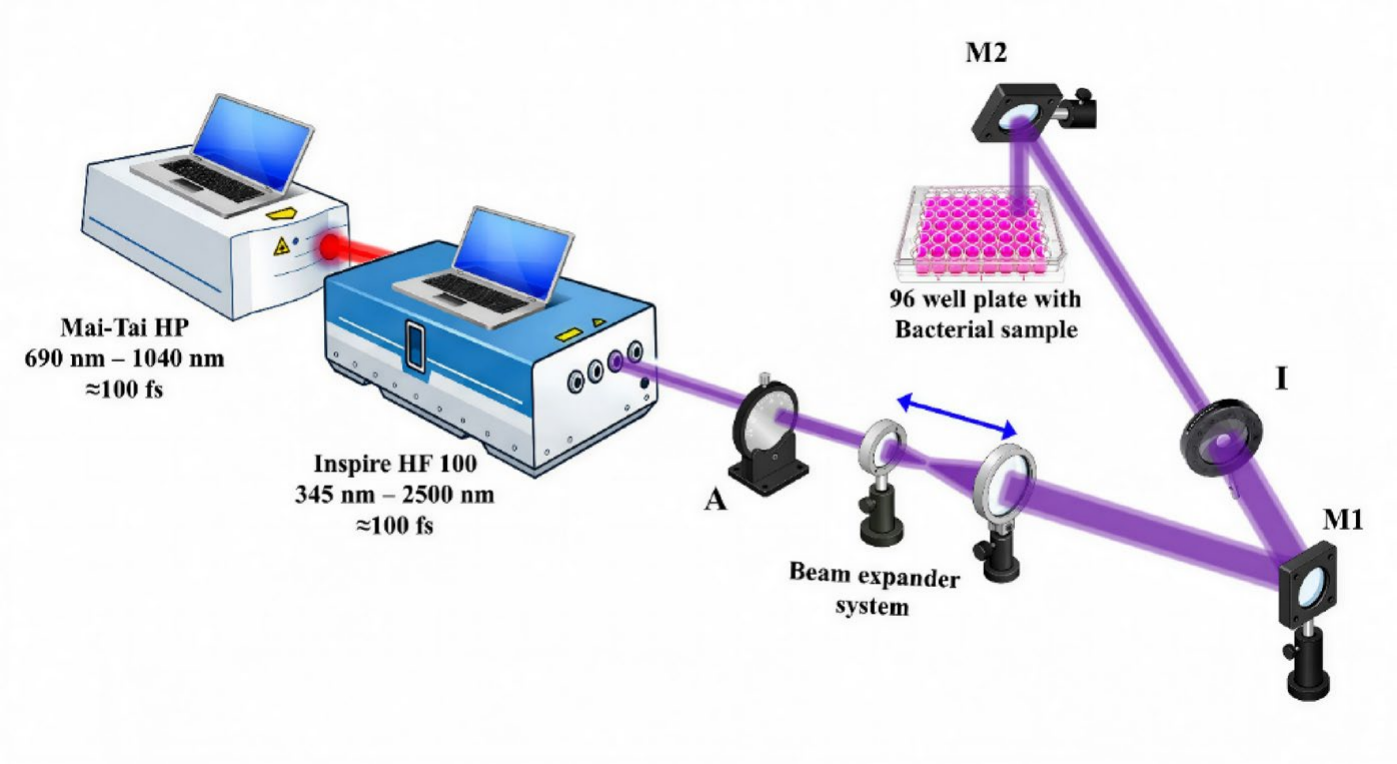
Schematic diagram of the experimental setup for the antimicrobial femtosecond laser treatment. A: Attenuator, M1, and M2: Highly reflective mirrors, I: Iris.

Femtosecond laser irradiation was performed using an optimized protocol: 400 nm wavelength, 100 fs pulses at 80 MHz repetition rate. The femtosecond laser pulses were emitted from the INSPIRE HF100 laser system (Spectra-Physics), which was pumped by a mode-locked femtosecond Ti: sapphire MAI TAI HP laser (Spectra-Physics). The spatial profile of the laser beam was a Gaussian distribution with a TEM00 spatial mode. The INSPIRE HF100 laser system was operated at the Second Harmonic Generator (SHG) mode to convert the wavelength of 800 nm from the MAI TAI HP into 400 nm. To ensure homogeneous irradiation of the bacterial cultures, the laser beam was precisely manipulated from the output aperture to reach the target area approximately 10 cm above the bacterial cultures with the help of a series of optical and optomechanical components, as illustrated in Fig. 1. Attenuator A was used to control the average laser power, which was measured using the Newport 843R power meter. A beam expander of two converging lenses was used to expand the initial laser beam’s diameter from 2 mm to 10 mm. Two highly reflective mirrors, M1 and M2, were used to direct the laser beam to the samples. The beam profile was shaped from its native Gaussian distribution to a semi-flat-top profile by selecting the central portion of the beam (6 mm) with an adjustable iris, ensuring uniform irradiation across the biofilm area. All irradiation experiments were performed at a controlled ambient temperature of 18–19 °C, below standard room/incubator temperature (~ 22–25 °C). This provided an additional thermal buffer below temperatures associated with bacterial heat stress (typically > 42 °C for many species) and increased the temperature gradient between the sample and its surroundings.

Two complementary irradiation modalities were used to vary average power and exposure duration independently, as shown in Table 1: (1) varying exposure time (15, 30, and 45 min) at a constant average power of 50 mW; and (2) varying average power (50, 100, and 150 mW) at a constant exposure duration of 15 min. To ensure accurate dosimetry, the average power ($\:{P}_{avg}$) was converted to irradiance (* I*) and fluence (* F*) using the beam area, $\:A=\pi\:{r}^{2}=0.283\:{\mathrm{cm}}^{2}$, calculated from the 6 mm spot diameter. Irradiance was obtained as * I* = $\:{P}_{avg}$/ * A*, total delivered energy as $\:{E}_{total}\:$= $\:{P}_{avg}$ × t (where t is the exposure duration), and fluence as * F* = $\:{E}_{total}$/ * A*, equivalently * F* = ($\:{P}_{avg}$/ * A*) × t. This design allowed the same three fluence levels to be reached through two independent parameter combinations, enabling assessment of whether the biological response depended on total fluence alone or also on irradiance and exposure duration individually.

**Table 1:** Irradiation parameters and calculated dosimetry at wavelength of 400 nm, 100 fs pulse duration, 6 mm spot diameter (Area = 0.283 cm²).

Sample name	$\:{\boldsymbol{P}}_{\boldsymbol{a}\boldsymbol{v}\boldsymbol{g}}$(mW)	t (min.)	$\:{\boldsymbol{E}}_{\boldsymbol{t}\boldsymbol{o}\boldsymbol{t}\boldsymbol{a}\boldsymbol{l}}$(J)	$\:\boldsymbol{I}$(W/cm²)	$\:\boldsymbol{F}$(J/cm²)
T15/P50	50	15	45	0.177	159
T30	50	30	90	0.177	318
T45	50	45	135	0.177	477
P100	100	15	90	0.354	318
P150	150	15	135	0.530	477

### Assessing femtosecond laser attenuation of bacterial biofilm formation

Overnight cultures of MRSA were diluted to 0.5 McFarland concentration, then 200 µL aliquots were dispensed into 96-well plates (Corning Costar). The wells containing bacterial samples were then exposed to a 400 nm femtosecond laser beam (6 mm diameter) for different exposure durations (15, 30, and 45 min). The 96-well plates were then incubated without shaking for 2, 4, and 6 h at 37 °C to allow biofilm formation. The culture media were carefully removed from the wells. Each well was washed once with 200 µl of phosphate-buffered saline (PBS) to remove non-adherent cells before assessment.

### Quantitative biofilm viability analysis (CFU assay)

To determine the number of viable bacterial cells within the biofilm, a colony-forming unit (CFU) assay was performed. After removal of non-adherent cells with the wash buffer, 200 µl of fresh PBS was added to each well. The adherent biofilm was then thoroughly detached by mechanical scraping and pipetting, and the resulting suspension was transferred to sterile microcentrifuge tubes. To ensure complete dissociation of the biofilm clusters into individual cells, the samples were sonicated for 2 min at a frequency of 20–40 kHz in an ice bath to prevent thermal damage. The suspension was then vortexed for 15 s. Serial ten-fold dilutions were prepared in PBS, and 10 µl aliquots of each dilution were plated onto nutrient agar. The plates were incubated at 37 °C for 24 h, after which the colonies were counted, and the results were expressed as CFU/ml.

### Biofilm biomass quantification (crystal violet assay)

The formed biofilms were air-dried for 15 min. Subsequently, 125 µl of 0.1% (w/v) crystal violet solution was added to each well and incubated at room temperature for 30 min. The excess stain was removed, and the wells were washed twice with 200 µl of PBS to ensure the removal of unbound dye. The plates were then air-dried for an additional 30 min. To solubilize the biofilm-bound stain, 200 µl of 95% ethanol was added to each well, followed by incubation for 20 min with gentle shaking. The resulting solution was transferred to a new plate, and the optical density was measured at 570 nm using a microplate reader (Multiskan™ FC).

### Biofilm metabolic activity assessment (MTT assay)

The plates were air-dried for 5 min, then the metabolic activity of the adherent biofilms was evaluated using the MTT assay (Sigma-Aldrich), where 100 µL of 5 mg/mL MTT solution in PBS was added to each well and incubated for 3 h at 37 °C. The formed formazan crystals were solubilized in 100 µL dimethyl sulfoxide (DMSO; Sigma-Aldrich) for 15 min, then a fraction of 70 µL was transferred to a new plate, and absorbance was measured at 570 nm using a microplate reader (Multiskan™ FC).

### Assay for femtosecond laser-mediated biofilm destruction

Mature non-irradiated biofilms were formed by extending the incubation period to 24 h in BHI broth. Excess broth was aspirated before femtosecond laser treatment. The biofilms were gently washed twice with sterile PBS to remove loosely attached cells, then the adherent biofilms were subjected to femtosecond laser irradiation at different average powers (50, 100, and 150 mW) for different exposure durations (15, 30, and 45 min). Immediately post-irradiation, quantitative biofilm viability was analyzed, biofilm biomass was evaluated using the crystal violet assay, and biofilm metabolic activity was quantified using the MTT assay as previously described and compared to non-irradiated controls.

### Scanning electron microscopy (SEM) analysis of biofilm ultrastructure

For high-resolution morphological analysis, biofilms were grown on 0.02 μm nylon sheets to observe the morphology of bacterial cells and biofilms after femtosecond laser treatment. The biofilms were cultured for 24 h, washed with PBS, and fixed with glutaraldehyde solution (2.5%) at room temperature. Afterwards, the nylon sheets were air-dried. Imaging was performed using a ZEISS Sigma 500 VP field-emission scanning electron microscope (FE-SEM). Dried specimens were mounted on aluminum stubs using carbon tape and sputter-coated with a 10 nm layer of gold/palladium (80:20) using a Quorum Q150T ES coater. The microscope was operated at an accelerating voltage of 6.00 kV with a working distance of approximately 5 mm. Micrographs were acquired using the secondary electron (SE2) detector at magnifications of 1,000x, 5000×, 10,000×, and 20,000× to examine femtosecond laser-induced structural modifications, including bacterial membrane damage and cellular debris distribution compared to non-irradiated controls.

### Quantitative analysis of quorum-sensing and biofilm-related gene expression using RT-qPCR

Using the Total RNA Isolation Kit (GeneDireX, Cat. No. NA017-0100), the total RNA was extracted from femtosecond laser-treated and control MRSA samples according to the manufacturer’s instructions. An on-column DNase I treatment was performed to eliminate genomic DNA contamination. The integrity of the RNA was confirmed by visualization on a 1% agarose gel. First-strand cDNA synthesis was performed from 1 µg of total RNA using the GScript First-Strand Synthesis Kit (GeneDireX, Cat. No. MB305-0050) with random hexamer primers, following the manufacturer’s protocol. Quantitative PCR was performed using the TOPreal™ One-step RT qPCR Kit (SYBR Green with low ROX) (Enzymomics). The reverse transcription reaction was carried out at 45 °C for 45 min, followed by enzyme inactivation at 70 °C for 15 min. For this experiment, the two-step protocol was employed, utilizing the synthesized cDNA. Reactions were prepared in triplicate in a total volume of 20 µL, containing 10 µL of TOPreal™ One-step RT qPCR Reaction MIX (with low ROX), 1 µL each of forward and reverse primers (10 µM; see Table S1 for sequences), 2 µL of cDNA template, and 6 µL of nuclease-free water. The reactions were run on a SaCycler-96 Real-Time PCR System with the following cycling conditions: initial denaturation at 95 °C for 10 min; followed by 45 cycles of denaturation at 95 °C for 10 s, and a combined annealing/extension at 60 °C for 45 s. A melting curve analysis was performed immediately after amplification from 60  to 95 °C to verify the specificity of the amplification products. The housekeeping genes *aroE and gyrB* were used for normalization. Relative gene expression was calculated using the ΔCq and log2FC methods.

### Statistical analysis

Statistical analysis was performed using R (version 4.3.1, R Foundation for Statistical Computing) and Python (version 3.11, Python Software Foundation). For comparisons, a one-way analysis of variance (ANOVA) was conducted, followed by pairwise comparisons using Tukey’s Post-hoc test. Data were presented as the mean ± standard error of the mean (SEM) from at least three independent biological replicates, each with technical triplicates. The effect size for group comparisons was calculated as Cohen’s “d” for pairwise comparisons, and eta-squared (η²) for the one-way ANOVA to quantify the proportion of total variance attributable to the experimental treatment. Correlation analyses between genes were assessed using Pearson’s correlation coefficient (“r”). For quantitative analysis of SEM images, images were contrast-enhanced using CLAHE, and biofilm coverage, cell density, matrix fraction, and structural damage were quantified by multi-level Otsu thresholding and distance-transform watershed segmentation. Statistical comparisons were performed using the two-sided Mann–Whitney U test against the untreated control group. Data visualization was generated using the *ggplot2* package in R, the *seaborn scikit-image*, and *matplotlib* libraries in Python. Statistical analysis was performed using *scipy.stats*, and *statsmodels* in Python, complemented by the stats package in R for specific implementations. Statistical significance thresholds were set at *p <* 0.05 (*),* p <* 0.01 (**),* p <* 0.001 (***), and *p <* 0.0001 (****).

## Results

Mechanical and biological interactions between bacteria and host cells, leading to the formation of biofilms, are a key element of bacterial infection because bacteria employ various virulence mechanisms that enable the progression of infection. Disruption of MRSA virulence factors can be investigated by subjecting the bacteria to sublethal doses of aPDT treatment, a dose previously determined to result in a 1 to 3 log_10_ reduction in colony-forming units CFU/mL of the pathogen^28,29,30,31,32,33^, leaving sufficient survivors to test the possible attenuation of MRSA adhesion and subsequent biofilm formation after different femtosecond laser treatments. In a recent study^34^, we checked the validity of the proposed parameters (wavelength of 400 nm, average power of 50, 100, and 150 mW, for exposure durations of 15, 30, and 45 min) as sublethal femtosecond laser doses while investigating the efficacy of femtosecond laser irradiation in reducing the adhesion and invasion capability of MRSA to different human cell lines.

### Evaluating the potential of femtosecond laser exposure for attenuating MRSA biofilm formation

**Fig. 2:**
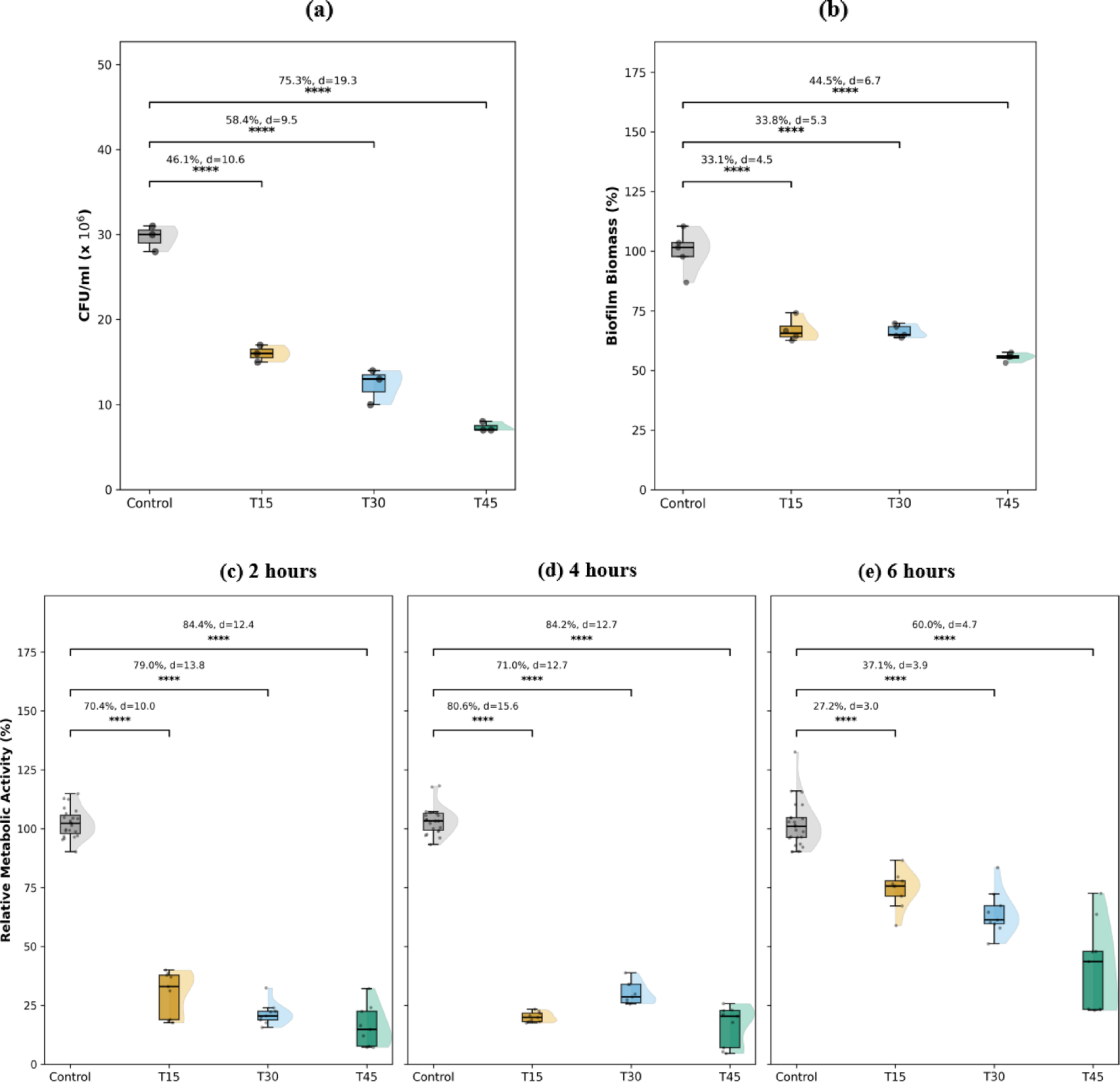
Comparative evaluation of MRSA biofilm formation after 400 nm femtosecond laser irradiation. Biofilms were evaluated after femtosecond laser treatment at a wavelength of 400 nm, and an average power of 50 mW for 15 min (T15), 30 min (T30), and 45 min (T45) at different biofilm maturation durations compared to a control. (**a**) MRSA biofilms Viability quantified as CFU*10^6^/mL after a 6-hour incubation period, (**b**) Biofilm biomass percentage after a 6-hour incubation period, (**c**), (**d**), and (**e**) relative metabolic activity after different incubation periods; 2, 4, and 6 hours, respectively. Data presented as boxplots (median, quartiles) with half-eye distributions and individual replicates (points). Statistical significance versus control was determined using ANOVA followed by Tukey’s test, **** *p <* 0.0001. Effect sizes are reported as percentage reduction and Cohen’s d.

To evaluate the ability of MRSA to form biofilms after femtosecond laser exposure at 400 nm, with average power of 50 mW, for 15 min (T15), 30 min (T30) and 45 min (T45) compared to the untreated control (X-axis), a definitive quantification of the bactericidal efficacy after a 6-hour incubation period was provided in Fig. 2a, quantified as colony-forming unit per milliliter, CFU/ml (Y-axis). In Fig. 2b, the potential of 400 nm femtosecond laser irradiation to prevent the establishment of MRSA biofilms was quantified as biofilm biomass percentage (Y-axis) after a 6-hour incubation period using the crystal violet (CV) assay. Furthermore, the physiological state of the surviving bacterial population was evaluated using the MTT assay, which measures dehydrogenase enzyme activity as a proxy for metabolic activity (Y-axis), after three distinct durations of biofilm maturation: 2 (Fig. 2c), 4 (Fig. 2d), and 6 h (Fig. 2e).

As shown in Fig. 2a, femtosecond laser treatment induced a profound, time-dependent reduction in the number of viable MRSA cells. A significant reduction of 46.1% (Cohen’s d = 10.6, *p <* 0.0001) was observed even after the shortest exposure of 15 min (T15), with the mean CFU/ml dropping from approximately $\:30\times\:{10}^{6\:}$in the control to $\:16\times\:{10}^{6}$. The bactericidal effect intensified as the exposure duration increased, with a 58.4% reduction at 30 min (T30; d = 9.5, *p <* 0.0001) and reaching a maximum reduction of 75.3% at 45 min (T45; d = 19.3, *p <* 0.0001). In Fig. 2b, the femtosecond laser treatment exerted a potent and highly significant inhibitory effect on early biofilm development. Even the shortest exposure of 15 min (T15) resulted in a substantial 33.1% reduction in biofilm biomass (Cohen’s d = 4.5, *p <* 0.0001). This inhibitory effect was further enhanced at 45 min, achieving a maximum reduction of 44.5% (T45; d = 6.7, *p <* 0.0001). These findings indicate that the total delivered energy, particularly when extended over longer durations, is a critical factor in the physical disruption of the MRSA biofilm matrix. In Fig. 2c-e, the femtosecond laser treatment demonstrated a powerful and statistically significant inhibition of biofilm metabolic activity at all tested femtosecond laser doses (T15, T30, T45) compared to the untreated control (One-way ANOVA, *p <* 0.0001). The anti-biofilm effect of the treatment was highly dependent on the maturation state of the biofilm, with the most marked effect observed at the earliest time point of 2 h, where the shortest exposure of 15 min (T15) reduced biofilm formation by 84.4% (Cohen’s d = 12.4), with 30 min and 45 min exposure showing similar potent reductions of 79.0% (T30; d = 13.8, *p <* 0.0001) and 70.4%, (T45; d = 12.4, *p <* 0.0001) respectively (Fig. 2c). This strong inhibitory effect was maintained at 4 h (Fig. 2d), and while somewhat attenuated, remained significant at 6 h (Fig. 2e), indicating that the initial treatment effectively cripples the bacterial ability to establish a mature biofilm.

**Fig. 3:**
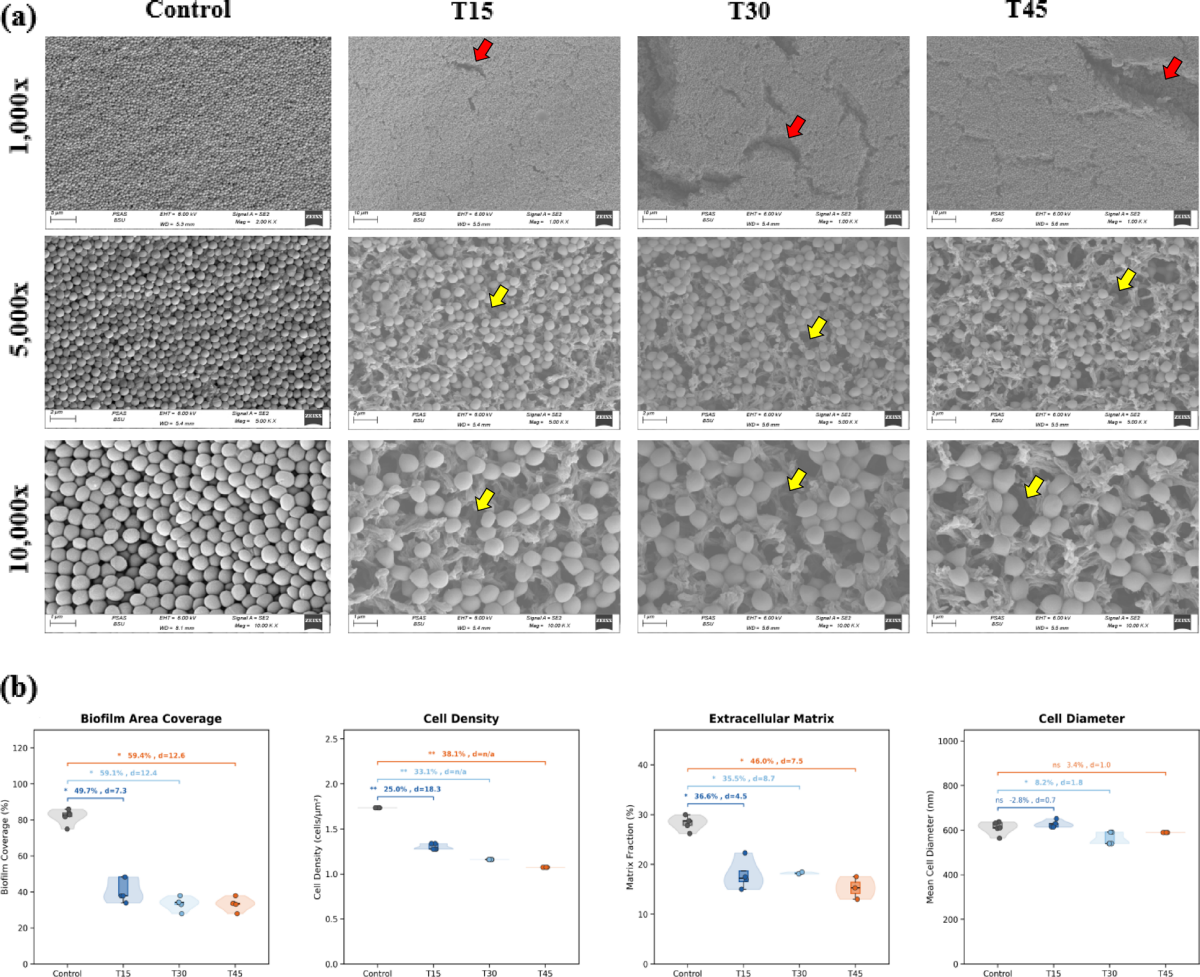
Quantitative Scanning Electron Microscope (SEM) images Analysis of MRSA biofilms after planktonic cells exposure to 400 nm, 50 mW femtosecond laser treatment for 15 min (T15), 30 min (T30), and 45 min (T45) compared to untreated control. (**a**) Images showing structural integrity captured at increasing magnifications: 1,000X (first row), 5,000 X (second row), and 10,000 X (third row). The control samples show intact, smooth, and characteristic spherical morphology, organized in clusters, whereas the femtosecond laser-treated samples display a clear disruption of the biofilm architecture, characterized by a substantial reduction in the extracellular polymeric substances matrix and the presence of disaggregated bacterial cells. (**b**) Quantitative SEM-derived parameters assessed: biofilm area coverage (%), bacterial cell density (cells/µm²), extracellular polymeric substance (EPS) matrix fraction (%), and mean cell diameter (nm). Data are presented as violin plots overlaid with box plots (median, IQR, and 1.5× IQR whiskers) and individual data points, each representing one SEM image. Percentage change relative to control and Cohen’s *d* effect size are indicated above each bracket. Statistical significance was determined by two-sided Mann–Whitney U test vs. control: *p < 0.05, **p < 0.01, ***p < 0.001, ****p < 0.0001; ns = not significant.

To gain further information on the mechanism behind the use of femtosecond laser aPDT to prevent biofilm formation, in Fig. 3a, we performed scanning electron microscopy (SEM) analysis on 24-hour MRSA biofilms formed following exposure of planktonic cells to femtosecond laser compared to control samples. The femtosecond laser treatment showed efficacy at preventing the initial attachment and growth of biofilms. Instead of a smooth, confluent layer, formed by the control group, femtosecond laser-treated biofilms show sparse, isolated clusters of cells, with a significant reduction in the amount of EPS matrix that would normally glue them together. At 1,000x magnification, the red arrows point to the site where the biofilm failed to form. At higher magnifications (5,000x and 10,000x), the yellow arrows highlight the empty spaces between cell clusters. To further evaluate the capacity of femtosecond laser irradiation to inhibit MRSA biofilm formation, Fig. 3b, presents four SEM-derived parameters; biofilm area coverage (%), cell density, extracellular matrix fraction (%), and mean cell diameter (Y-axes) after femtosecond laser exposure at 400 nm with an average power of 50 mW for three exposure durations: 15 min (T15), 30 min (T30), and 45 min (T45), compared to the untreated control (X-axis). As evident, the femtosecond laser treatment induced a dramatic reduction in biofilm area coverage (%) with a 59.4% reduction at T45 (*p* < 0.01). Matrix fraction (%) also dropped by 46.0% at T45 (d = 7.5, *p* < 0.05), while the data for cell density (cells/µm²) shows a significant and dose-dependent decline as well, reaching a 38.1% reduction at T45 (*p* < 0.01). The mean cell diameter remained relatively stable, suggesting that the femtosecond laser treatment does not cause bulk cellular shrinkage but rather targets specific membrane and matrix components, leading to a loss of structural integrity without necessarily changing the overall cell size.

### Uncovering the transcriptional and regulatory network changes associated with femtosecond laser-based aPDT

**Fig. 4:**
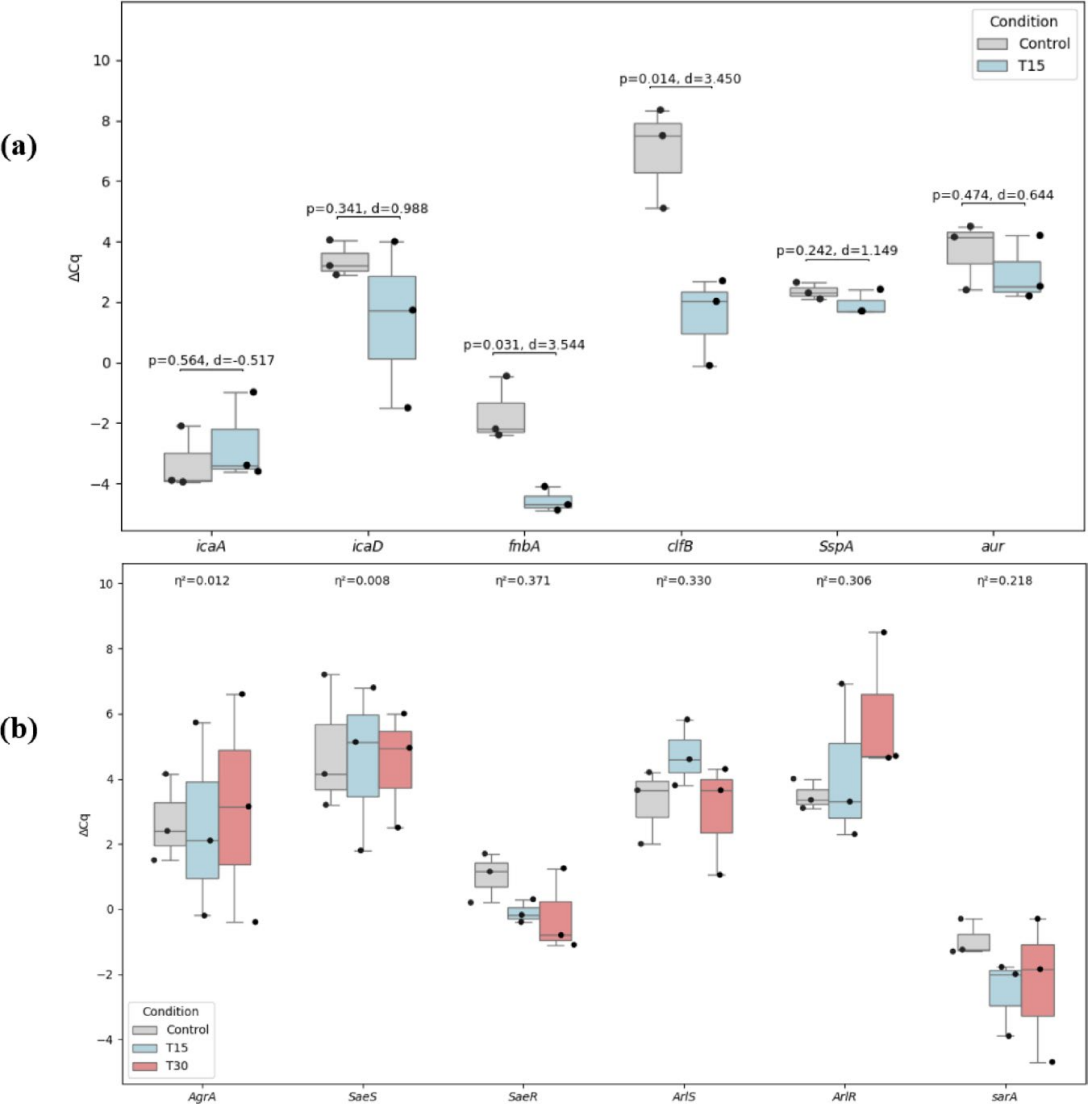
Analysis of MRSA virulence and quorum-sensing relative genes expression following femtosecond laser treatment compared to the control sample. (**a**) Normalized Cq values (ΔCq) of genes involved in adhesion (*fnbA* and *clfB*), biofilm matrix synthesis (*icaA* and *icaD*), and biofilm dispersion/immune evasion (*SspA* and *aur*). (**b**) Expression of global regulators of quorum-sensing and virulence (*AgrA*, *SaeR*, *ArlS*, *ArlR*, and *sarA*). The light blue boxplots (L15) represent the 15-minute femtosecond laser treatment, the red boxplots (T30) represent the 30-minute femtosecond laser treatment, both at an average power of 50 mW, at a wavelength of 400 nm, compared to the grey boxplots (Control). Statistical significance for panel (**a**) was determined using Student’s unpaired t-test, with p-values and Cohen’s d values displayed above each gene. Statistical analysis for panel (**b**) was performed using a one-way ANOVA, with $\:\eta\:$^2^ values indicating the effect size of the different treatment conditions on gene expression.

In Fig. 4, we performed RT-qPCR analysis on key virulence and quorum-sensing genes in MRSA to investigate the molecular mechanism behind the observed attenuation of MRSA biofilm formation ability. The relative gene expression (Y-axis) as normalized Cq values (ΔCq) of key virulence factors was quantified after exposure to femtosecond laser treatment (T15: 400 nm, 50 mW for 15 min) compared to the untreated control. The assessed genes (X-axis) encode critical virulence factors involved in host-pathogen interactions. The figure is divided into two panels: panel (a) and panel (b). Panel (a) focuses on genes associated with adhesion (*fnbA* and *clfB*), biofilm matrix synthesis (*icaA* and *icaD*), and biofilm dispersion and immune evasion (*SspA* and *aur*). Panel (b) investigates the expression of genes functioning as global regulators of quorum sensing and virulence (*AgrA*, *SaeS*, *SaeR*, *ArlS*, *ArlR*, and *sarA*), where an increased exposure duration of 30 min (T30) is used to explore the dose-dependent modulation of the regulatory network.

As shown in panel (a), the T15 treatment had a significant effect on the expression of the adhesion genes, specifically *clfB* and *fnbA* (lower ΔCq = higher expression), suggesting a profound effect on the initial stages of biofilm formation. Furthermore, panel (b) shows the differential and complex impact of the T15 and T30 treatments on the global regulatory network. The varied and seemingly erratic expression patterns across the T15 and T30 groups, particularly for *SaeR*, *ArlS*, *ArlR*, and *sarA*, suggest a significant dysregulation of the quorum-sensing and virulence pathways, a phenomenon that is further explored through correlation analysis in the next figure. The large effect sizes ($\:\eta\:$^2^ > 0.2) observed for these genes underscore the potent, complex, modulatory effect of the higher femtosecond laser dose.

**Fig. 5:**
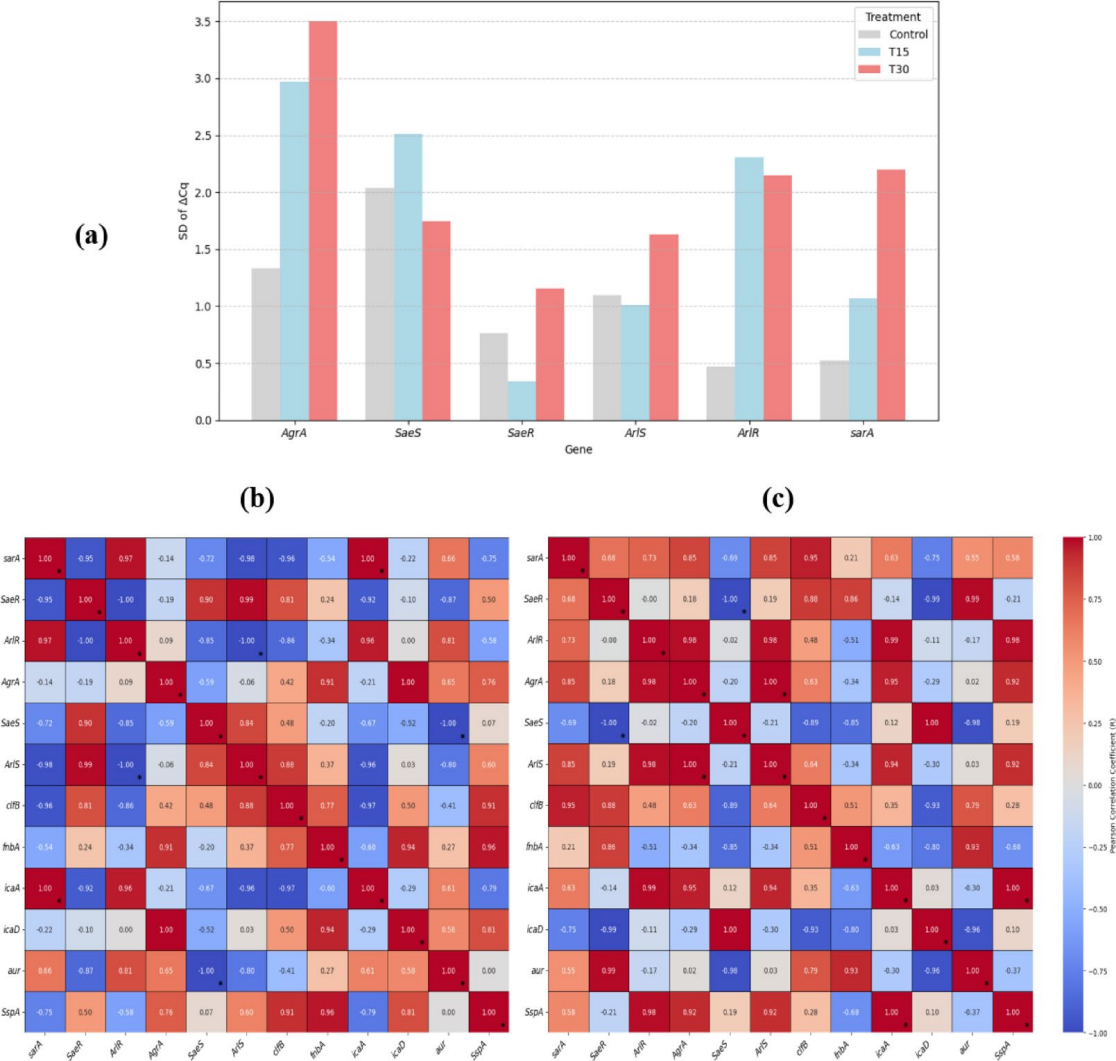
Expression variability and gene-gene correlation analysis of the investigated virulence and quorum-sensing genes in MRSA after exposure to femtosecond laser irradiation. (**a**) Shows the expression variability of the global regulators (*AgrA*, *SaeS*, *SaeR*, *ArlS*, *ArlR*, and *sarA*) in the control (grey), T15 (light blue), and T30 (red) treatment groups, all at an average power of 50 mW. Panels (**b**) and (**c**) are heatmaps illustrating the Pearson correlation coefficient (r) between the expression levels of all investigated genes (adhesion: fnbA, *clfB*; biofilm matrix: *icaA*, *icaD*; dispersion/immune evasion: *SspA*, *aur*; global regulators: *AgrA*, *SaeS*, *SaeR*, *ArlS*, *ArlR*, and *sarA*). Panel (**b**) represents the native correlation network in the untreated control sample. Panel (**c**) represents the restructured correlation network after the T15 treatment (15 min at 50 mW). The color scale indicates the strength and direction of the correlation, with red representing a strong positive correlation and blue representing a strong negative correlation. An asterisk (*) indicates a statistically significant correlation (*p <* 0.05).

Beyond simple up-regulation or down-regulation of specific genes, the femtosecond laser treatment appears to destabilize the entire quorum-sensing (QS) regulatory network. In Fig. 5a, the standard deviation (SD) of ΔCq (Y-axis) for each quorum-sensing gene (X-axis) is shown. While the earlier boxplots show changes in the average expression, this bar chart shows changes in the *consistency* of expression. A higher bar means more variability (less precise regulation) between the three biological replicates. As is evident, femtosecond laser treatments, particularly the T30 dose, induce a significant increase in the expression variability for most of the QS regulators (especially *AgrA*, *SarA*, and *ArlR*), supporting the hypothesis of a highly dysregulated state. Furthermore, the effect of the femtosecond laser treatment on the underlying gene regulatory network is shown in Fig. 5b, c. Panel (b) represents the native gene-gene correlation network in the untreated control, showing the baseline relationships between all investigated genes. In contrast, panel (c) demonstrates the restructured correlation network after the T15 treatment. A clear and profound disruption of the native regulatory relationships is observed, with many strong correlations in the control being attenuated or reversed in the T15 group.

**Fig. 6:**
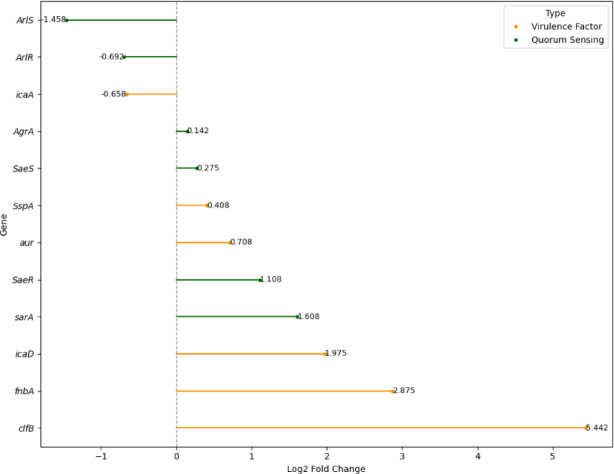
Ranking of gene expression changes after femtosecond laser irradiation. The Log2 Fold Change (LFC) of all the investigated genes, comparing the femtosecond laser treatment (T15 at 50 mW) to the untreated control. The genes are color-coded by type: Virulence Factor (Orange) and Quorum Sensing (Green). The LFC values are displayed next to each bar, clearly ranking the magnitude of the femtosecond laser-induced change in gene expression.

The overall effects of the femtosecond laser treatment on the gene expression profile were plotted in Fig. 6 using a lollipop plot for Log2 Fold Change (LFC) value (X-axis) of all the investigated genes (Y-axis), providing a clear ranking of the most affected virulence factors and quorum-sensing genes. Genes with positive LFC values are upregulated, meaning their expression increased, while those with negative values are downregulated, indicating decreased expression.

We found that the adhesion genes *clfB* (LFC = 5.442 ~ 43-fold increase) and *fnbA* (LFC = 2.875 ~ 7-fold increase), as well as the biofilm synthesis gene *icaD* (LFC = 1.975 ~ 3.9-fold increase), showed the most pronounced upregulation, suggesting a strong compensatory response to the femtosecond laser treatment. Conversely, the quorum-sensing regulators *ArlS* (LFC = -1.45 ~ 2.8-fold decrease) and *ArlR* (LFC = -0.692 ~ 1,6-fold decrease), as well as the biofilm synthesis gene *icaA* (LFC = -0.658 ~ 1.58-fold decrease), showed significant downregulation. This differential regulation highlights the potent ability of the femtosecond laser to disrupt the fine-tuned balance within the virulence network.

**Fig. 7:**
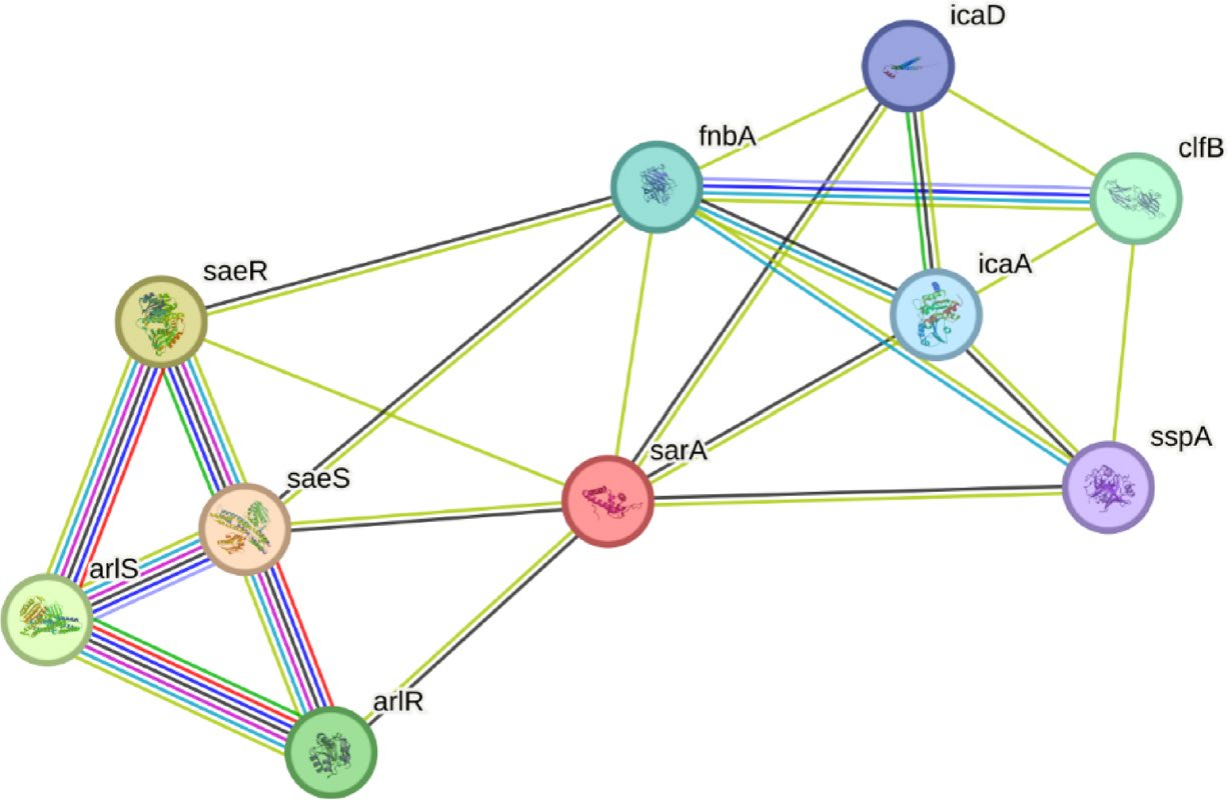
Protein-Protein Interaction (PPI) network of the investigated genes generated using the STRING database (version 12.0) for *Staphylococcus aureus*. The network visually illustrates the central role of the Quorum-sensing regulators (*sarA*, *SaeR*, *SaeS*, *ArlS*, *ArlR*) in controlling the expression of the Virulence Factors (*fnbA*, *clfB*, *icaA*, *icaD*, *SspA*).

To understand the biological context of these changes, we performed a pathway analysis using the STRING database. The resulting network in Fig. 7 reveals known Protein-Protein Interactions (PPI) between our target genes. The network clearly shows the central role of the quorum-sensing regulators, such as *sarA*, and the two-component system *SaeRS*, in controlling the expression of the adhesion and biofilm genes, thereby suggesting that the femtosecond laser-induced dysregulation of these central nodes could be the primary mechanism for the observed anti-virulence effect.

### Exploring the effects of different femtosecond laser treatments in eradicating pre-formed MRSA biofilms

**Fig. 8:**
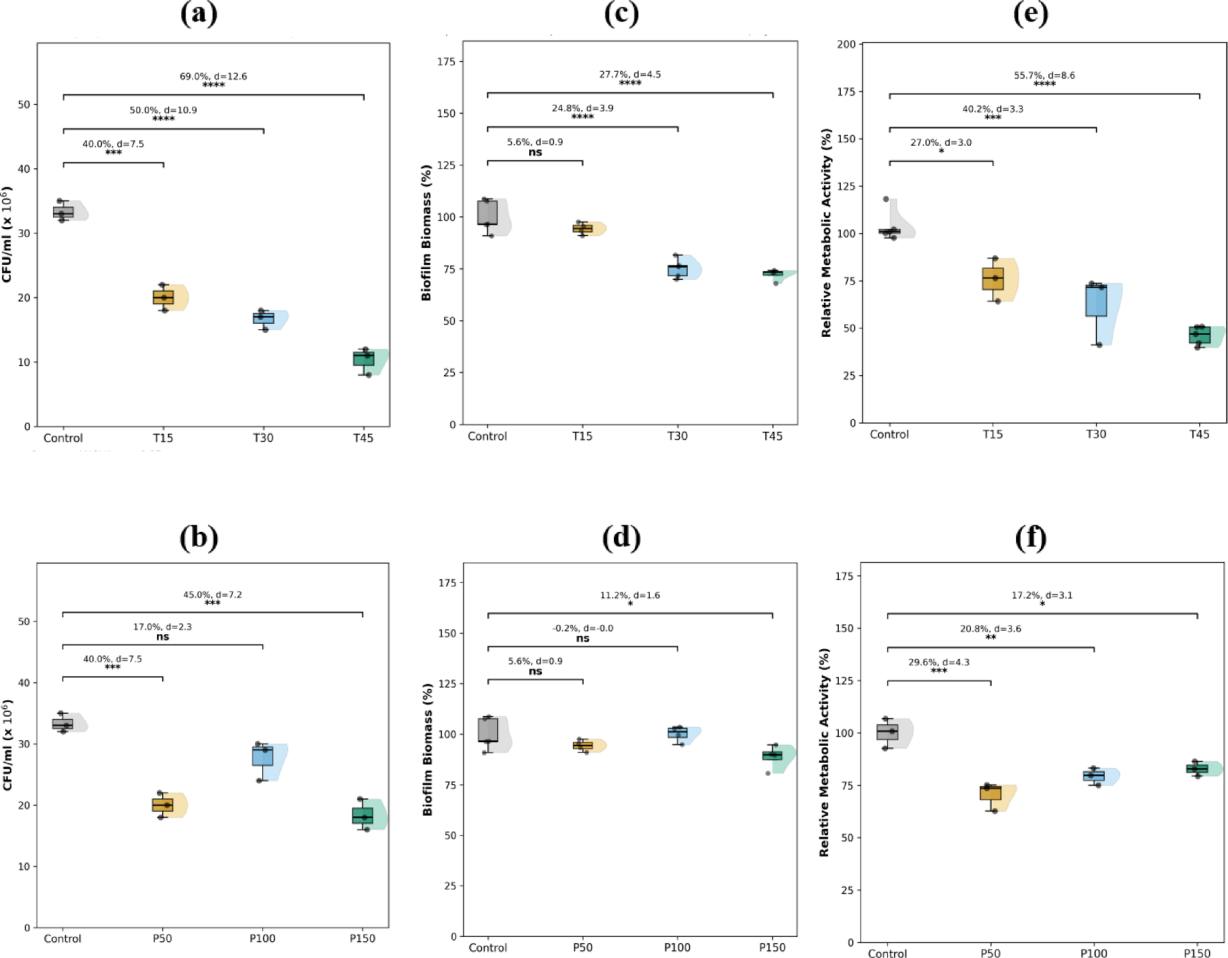
Comparative evaluation of 24-hour preformed MRSA biofilms destruction after 400 nm femtosecond laser treatment. Varying two different laser parameters: exposure durations (T15; 15 min, T30; 30 min, and T45; 45 min, at a constant average power of 50 mW), and average power (P50; 50 mW, P100; 100 mW, and P150; 150 mW, at a constant exposure duration of 15 min) compared to the untreated control. (**a**) and (**b**) Shows the Biofilm Viability quantified as CFU*10^6^/mL, (**c**) and (**d**) Biofilm biomass percentage, while (**e**) and (**f**) represent relative metabolic activity of the biofilms. Data are presented as boxplots (median, quartiles) with half-eye distributions and individual replicates (points). Statistical significance versus control was determined using ANOVA followed by Tukey’s test, with significance levels indicated by asterisks (* *p <* 0.05, ** *p <* 0.01, *** *p <* 0.001). Effect sizes are reported as percentage reduction and Cohen’s d.

In addition to our investigation into biofilm prevention, we next investigated the ability of the 400 nm femtosecond laser to destroy established, mature MRSA biofilms. As shown in Fig. 8, the destruction of preformed 24-hour MRSA biofilms was assessed comparatively by varying two different femtosecond laser parameters: (1) increasing exposure durations from 15 (T15), to 30 (T30), and 45 min (T45), at a constant average power of 50 mW; (2) increasing the average power from 50 (P50), to 100 (P100), and 150 mW (P150), while maintaining a constant exposure duration of 15 min compared to the untreated control (X-axis). In Fig. 8a, b, the bactericidal efficacy of the two modalities was quantified as colony-forming unit per milliliter, CFU/ml (Y-axis). In Fig. 8c, d, the reduction of biofilm biomass percentage (Y-axis) was assessed. While in Fig. 8e, f, the physiological state of the surviving bacterial population was evaluated using the MTT assay, which measures dehydrogenase enzyme activity as a proxy for metabolic activity (Y-axis).

As evident in Fig. 8a, the treatment induced a significant and dose-dependent reduction in viable cells with a linear increase in bactericidal efficacy with exposure time. A significant reduction of 40% was observed at 15 min (T15; d = 4.8, *p <* 0.001), which intensified to 50% at 30 min (T30; d = 5.5, *p <* 0.001) and reached a remarkable maximum of 69% at 45 min (T45; d = 8.7, *p <* 0.0001). In contrast, Fig. 8b showed less consistent results, with P100 being non-significant (17% reduction), P150 achieving a 45% reduction (d = 7.2, *p <* 0.001), and P50 achieving a 40% reduction (d = 7.5, *p <* 0.001). In Fig. 8c, the treatment resulted in a significant biomass reduction: 24.8% at 30 min (T30; d = 3.9, *p <* 0.0001) and reaching a maximum of 27.7% at 45 min (T45; d = 4.5, *p <* 0.0001). While in Fig. 8d, a significant reduction of 11.2% (Cohen’s d = 1.6, *p <* 0.05) was achieved only at the highest power of 150 mW (P150), whereas lower powers (P50 and P100) showed negligible effects on the total biomass. Figure 8e shows a profound and consistent reduction in biofilm metabolic activity, reaching a maximum reduction of 55.7% with a large effect size (d = 8.6, *p <* 0.0001) at T45. Conversely, increasing the average power (Fig. 8f) yielded a less pronounced and less consistent anti-biofilm effect, with the maximum reduction of 29.6% (d = 4.3, *p <* 0.001) observed at P50. This indicates that even the cells that evade immediate death (as measured by CFU) are metabolically crippled, further compromising the biofilm’s ability to maintain its structural integrity and virulence.

**Fig. 9:**
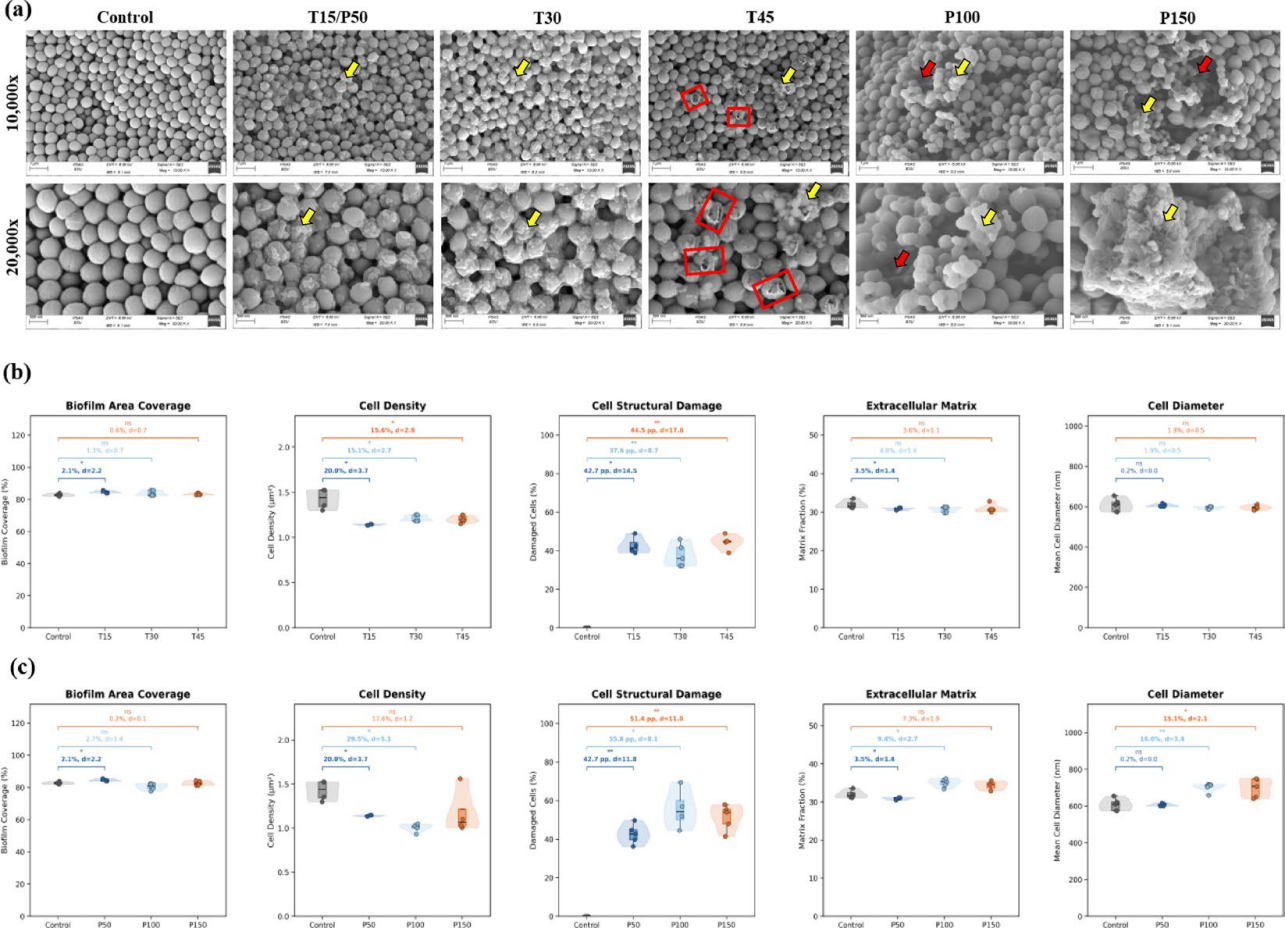
Quantitative Scanning Electron Microscope (SEM) images analysis visualizing the structural destruction of preformed 24-hour MRSA biofilms after 400 nm femtosecond laser treatment. (**a**) Shows the structural changes in MRSA biofilms induced by different femtosecond laser treatments captured at increasing magnifications: 10,000x (first row), and 20,000x (second row). The control samples show intact, smooth, and characteristic spherical cell morphology, organized in dense clusters. In the samples treated, the red boxes highlight areas of clear cell rupture and lysis, the yellow arrows point to cell membrane wrinkling, minor deformities, and surface damage, while the red arrows highlight large, amorphous clumps of fused or melted material, suggestive of a photothermal effect. (**b**) Quantitative SEM-derived parameters assessed: biofilm area coverage (%), bacterial cell density (cells/µm²), extracellular polymeric substances (EPS) matrix fraction (%), and mean cell diameter (nm) following femtosecond laser irradiation at 50 mW for 15, 30, or 45 min (T15, T30, T45) compared to an untreated control. (**c**) Quantitative SEM analysis of MRSA biofilms following femtosecond laser irradiation at 50, 100, or 150 mW for a fixed duration of 15 min (P50, P100, P150) compared to untreated control. Data are presented as violin plots overlaid with box plots (median, IQR, and 1.5× IQR whiskers) and individual data points, each representing one SEM image. Percentage change relative to control and Cohen’s *d* effect size are indicated above each bracket. Statistical significance was determined by two-sided Mann–Whitney U test vs. control: *p < 0.05, **p < 0.01, ***p < 0.001, ****p < 0.0001; ns = not significant.

To visually confirm the destructive effect of the femtosecond laser on preformed 24-hour MRSA biofilms, in Fig. 9a we performed SEM imaging following the induced structural changes after exposure to the two distinct treatment modalities: increasing the exposure duration (T15, T30, and T45) at a constant average power of 50 mW, and increasing the average power (P50, P100, and P150) at a constant exposure duration of 15 min. As shown, the Control sample displays a perfectly healthy biofilm, characterized by uniform, tightly packed *S. aureus* cocci encased in a smooth, extensive ECM. At T15/P50, the damage is visible but relatively minor, with the yellow arrow pointing to early signs of cell membrane wrinkling and slight disruption to the smooth ECM. At T30, the damage became more obvious, with the yellow arrows highlighting more pronounced cell deformation and the beginning of matrix degradation, resulting in the loss of the biofilm’s confluent appearance. The damage became pronounced and widespread at T45, with red boxes highlighting areas of clear cell rupture and lysis, and yellow arrows pointing to significant surface damage. The P100 and P150 images reveal a different, more severe type of damage. The red arrows point to large, amorphous clumps of fused or melted material, which is highly suggestive of a photothermal effect where the high power has induced thermal damage to the biofilm matrix and the cells within it. This visual evidence strongly supports the conclusion that increasing the duration of femtosecond laser exposure induced a more controlled, anti-virulence-based destruction, while increasing the power induced a less effective, heat-related damage mechanism.

To further assess the capacity of femtosecond laser irradiation to destroy pre-formed MRSA biofilms, Fig. 9b, presents five quantitative SEM-derived parameters; biofilm area coverage, cell density, proportion of damaged cells, extracellular matrix fraction, and mean cell diameter (Y-axes) after femtosecond laser exposure at 400 nm with an average power of 50 mW for three irradiation durations: T15; 15 min, T30; 30 min, and T45; 45 min (X-axis), while Fig. 9c, illustrates the same five SEM-derived parameters (Y-axis) following femtosecond laser irradiation at 400 nm for a fixed duration of 15 min at three power levels: P50; 50 mW, P100; 100 mW, and P150; 150 mW (X-axis), compared to the untreated control. As evident, biofilm area coverage (%), matrix fraction (%) and mean cell diameter remained relatively stable, while cell densities (cells/µm²) showed reduction by approximately ~ 15–30%, as well as the cell structural damage (%) significantly increased by approximately ~ 37–56%, reaching a peak of approximately 55.8% at P100 (d = 8.1, *p* < 0.05) and maintaining high levels in the T45 and P150 groups.

**Fig. 10:**
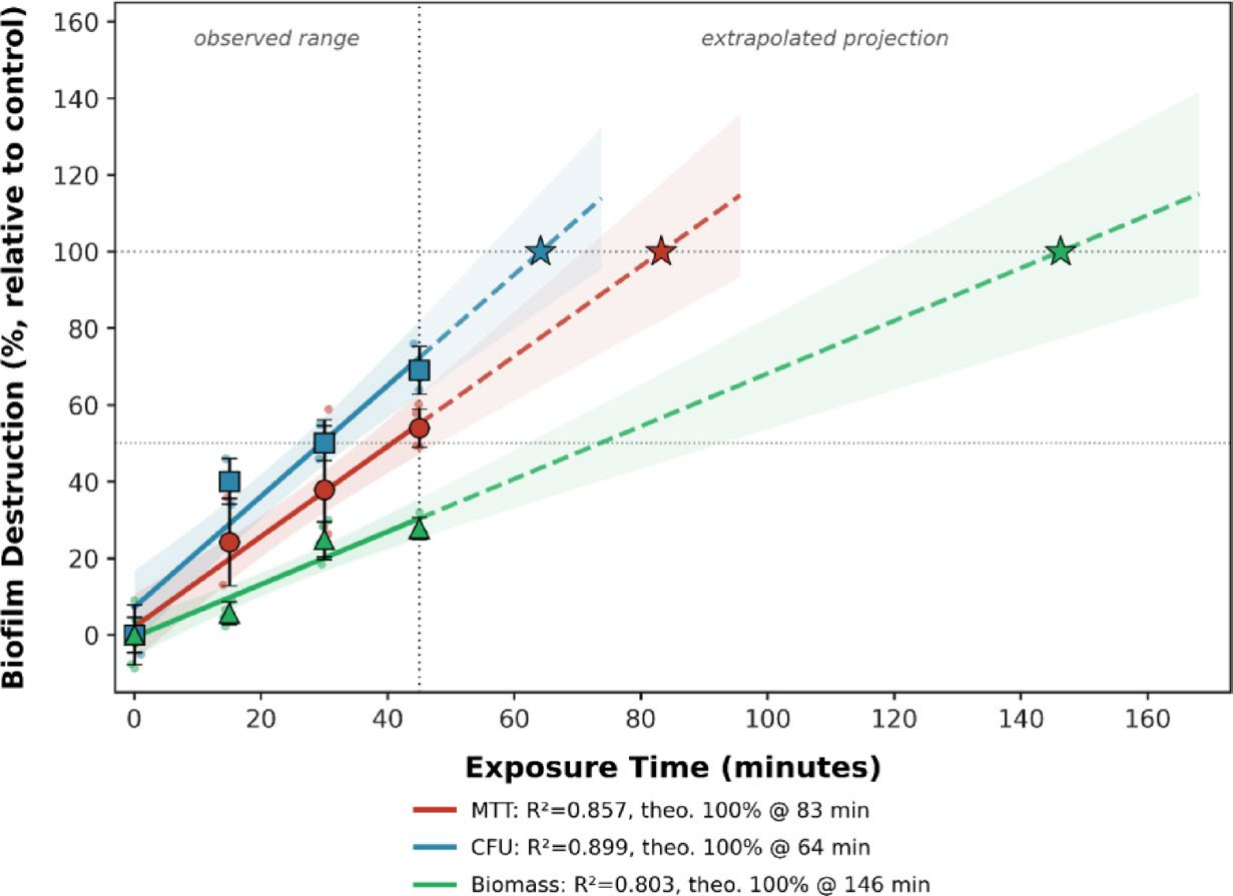
Multi-assay kinetics analysis of time-dependent femtosecond laser-induced biofilm destruction. Biofilm destruction percentage calculated from the biofilm viability/CFU assay (blue line), biofilm metabolic activity/MTT assay (red line), and biofilm biomass (green line) was represented as data (Mean ± SD) at each time interval. The data demonstrates a steady, linear, incremental increase in destruction, with a high coefficient of determination (R²).

These investigations clearly show the high dependence of the femtosecond laser-induced anti-biofilm effect on the treatment parameters, with the 45-minute 50 mW treatment standing out as the most effective modality for eradicating established MRSA biofilms. To elucidate the kinetics of MRSA biofilm eradication by femtosecond laser, in Fig. 10, the linear regression analyses were done to quantify the relationship between biofilm destruction (Y-axis) and exposure duration (X-axis). This confirms a strong linear correlation between treatment duration and biofilm eradication.

## Discussion

The pathogenicity of *S. aureus* is governed by a highly dynamic network, critically dependent on the coordinated expression of a diverse array of virulence factors. A central element of this pathogenicity is the ability to transition between a planktonic state, initial adhesion, the formation of a protective biofilm, and eventual biofilm dispersal to seed new infections^35^. This sequential process of biofilm formation and disruption provides several points for therapeutic interventions. Given the well-known inability of antibiotics to penetrate deep into the biofilm matrix, and their ineffectiveness against most MDR bacterial pathogens, new approaches are needed^36^. Laser-based aPDT is a promising alternative for combating these bacterial infections^37^.

The antimicrobial efficacy of 400 nm irradiation is largely attributed to the excitation of endogenous porphyrins, such as coproporphyrin III, which are naturally synthesized by *S. aureus.* These porphyrins possess a strong absorption band (Soret band) near 400 nm ^28,30^. Upon excitation, they facilitate the onsite generation of cytotoxic reactive oxygen species (ROS) resulting from the interplay between the laser radiation and compounds within the cells, transforming light energy to chemical energy via two concurrent photochemical processes^38,39^: (1) electron transfer reactions (type I), which generates hydrogen peroxide, superoxide, or hydroxide radicals, and (2) energy transfer reactions (type II), which produce highly reactive singlet oxygen (^1^O_2_)^40,41^. The resulting oxidative stress produced by these ROS eventually leads to cell death^42,43,44^. To rule out photothermal effects, our experimental conditions were optimized to minimize thermal effects as reported in prior studies^45,46^ and even with IR irradiation^47^. While direct ROS quantification was not performed in this study, the observed wavelength-dependent efficacy and the minimal thermal contribution are highly consistent with an endogenous aPDT mechanism^48,49,50^. When bacterial pathogens are subjected to sublethal doses of aPDT, the consequent oxidative stress within the bacterial cells may not be sufficient to kill the bacteria, but it can still affect microbial virulence factors and their expression^21,26,51^. This shift away from cell killing towards targeting virulence factors is particularly relevant considering the challenges posed by polymicrobial biofilms^52^. Therefore, the development of comprehensive antivirulence approaches that disturb the fundamental processes of adhesion, biofilm formation, and quorum sensing is a vital research area.

To provide a definitive quantification of the bactericidal efficacy of 400 nm femtosecond laser irradiation, MRSA viability was assessed after 6 h incubation of the irradiated samples compared to the control (early biofilm formation). As illustrated in Fig. 2a, the femtosecond laser treatment induced a profound, time-dependent reduction in the number of viable MRSA cells. The biofilm is not merely a collection of bacteria; it is a complex community protected by an EPS (polysaccharides, proteins, and eDNA). This matrix is the primary driver of antimicrobial resistance, acting as a physical and chemical shield^11^. In Fig. 2b, the femtosecond laser treatment exerted a potent and highly significant inhibitory effect on early biofilm development (biofilm biomass; the bacterial cells and the self-produced EPS matrix) across all tested exposure durations, with 44.5% reduction at the longer exposure duration (T45). Furthermore, the biofilm’s metabolic activity was quantified by the MTT assay in Fig. 2c-e, which revealed a massive and statistically significant reduction in biofilm viability, ranging from approximately 27% to 84% compared to the untreated control following femtosecond laser treatment across all investigated incubation periods (2 h, 4 h, and 6 h), underscoring its potent antibiofilm effect. As seen, the efficacy of the treatment was strongly dose-dependent, especially at the earlier stages of biofilm development (2 h and 4 h), with longer exposure durations (T45) resulting in the greatest reduction in biofilm viability. While the effect persisted at the 6 h time point, its effectiveness was reduced. This observation can probably be explained by the control biofilms entering a more mature, stationary phase where the metabolic activity decreases naturally, consequently limiting the sensitivity of the MTT assay. Furthermore, less-structured biofilms are relatively more susceptible to photo-treatment than the mature biofilm^53^. Nevertheless, the effect of the femtosecond laser remained statistically significant, confirming the sustained benefit of the treatment.

The potent inhibition of MRSA biofilm formation by 400 nm femtosecond laser irradiation was physically evidenced by the SEM micrographs and their corresponding quantitative analysis in Fig. 3, indicating a catastrophic failure in biofilm establishment with only sparse, isolated cell clusters visible, which provides a direct visual confirmation of the femtosecond laser’s potent anti-adhesion and anti-biofilm-formation capability. The clear lack of an ESP matrix confirms that the biofilm process was dysfunctional. In Fig. 3b, the quantitative SEM analysis demonstrated the effect of 400 nm femtosecond irradiation with a dose-dependent reduction in biofilm area coverage (%), and extracellular matrix fraction (%) compared to the control. This physical clearance of the surface correlates perfectly with the 44.5% reduction in total biomass observed in the crystal violet assay, as well as our molecular results, specifically the downregulation of the *icaA* and the *agr* quorum-sensing system, as discussed later. The loss of cell density is a direct physical manifestation of the 75.3% reduction in viability observed in the CFU assay. These results demonstrate that the femtosecond laser treatment effectively interferes with the initial stages of MRSA colonization and surface attachment, providing a robust preventative strategy against biofilm formation.

This study was intended to provide an analysis of the regulatory network that governs the biofilm lifecycle, which necessitated the selection of target genes representing each critical phase: adhesion/formation, global regulation, and dispersion. The initial establishment of infection relies on the capacity of MRSA bacteria to adhere to host tissues and surfaces. This process is primarily mediated by the Microbial Surface Components Recognizing Adhesive Matrix Molecules (MSCRAMMs), such as clumping factors B (*clfB*), which encode for proteins that bind to fibrinogen and promote clumping of bacteria, as well as allowing evasion of host immune defenses, and the fibronectin-binding proteins A **(***fnbA***)**, which facilitate binding to ESP matrix proteins like fibronectin^54^. After adhesion, the shift to a biofilm community is crucial for chronic infection and antibiotic resistance to take place. We selected the intercellular adhesion genes **(***icaA* and *icaD***)** as key indicators for biofilm formation, given their essential role in synthesizing the polysaccharide intercellular adhesin (PIA) specifically, poly-N-acetylglucosamine (PNAG), the crucial “glue” for cell-to-cell adhesion in a mature biofilm^55,56^. The final, yet equally critical, step of the biofilm lifecycle is dispersion, which enables bacteria to escape from the established biofilm and spread to new sites, often leading to acute systemic infections. This process is frequently mediated by the degradation of the biofilm matrix through secreted proteases^35^. Therefore, we included the genes for two major extracellular proteases, Serine Protease **(***SspA***)** and Aureolysin **(***aur***)**, as markers for biofilm dispersion. Both *SspA* and *Aur* are well-characterized extracellular proteases that function by degrading protein components of the biofilm matrix, thereby promoting the release of planktonic cells^57,58^.

The expression of these adhesion, biofilm-associated, and dispersion-related genes is precisely controlled by a complex network of global regulatory systems, which act as molecular switches in response to environmental cues and population cell density. To investigate this regulatory control, we selected representative genes from the four main regulatory systems operating in *S. aureus*: *Agr* (accessory gene regulator), *Sae* (*S. aureus* exoprotein expression), *Arl* (adaptive response to stress and host defense), and *SarA* (*Staphylococcus* accessory regulator A)^59^. The *Agr* system is a classic quorum-sensing mechanism, which is known to inversely regulate surface adhesins while promoting the expression of secreted virulence factors and biofilm dispersal^60^. The *Sae* and *Arl* genes further allowed us to investigate the influence of two-component systems that respond to extracellular signals and cell wall stress, respectively^61,62^. The *SarA* system acts as a global transcriptional regulator, controlling a large regulon that includes genes for adhesion, biofilm, and toxin production^63^. The combined analysis of these four systems provided a comprehensive framework for understanding the hierarchical control over the entire virulence cascade.

Strikingly, the gene expression data revealed an unexpected paradoxical response. 400 nm femtosecond laser treatment reveals a pattern of network-wide shifts in gene-gene correlations, providing a preliminary model for how femtosecond laser irradiation might disrupt the regulatory circuitry governing biofilm formation. On one hand, the treatment triggered a massive transcriptional upregulation of genes for the initial surface attachment, namely *clfB* and *fnbA*, as shown in Fig. 4a, where the large positive Cohen’s d indicates a very strong effect. However, these observed transcriptional changes did not translate into a corresponding phenotypic change, a classic consequence of severe stress^64^, as illustrated by our experiments in a recently published paper where we investigated the ability of 400 nm femtosecond laser treatment to attenuate the adhesion ability of MRSA to different host cell lines. In that study, we found the adhesion ability was severely modulated^34^, and this also aligns with our current results shown in Fig. 2.

In Fig. 4b, the femtosecond laser appeared to turn the MRSA regulatory machinery against itself, leading to a failed and abortive virulence program. The high variability (large boxes and spread of points) means the response was not consistent across all replicates, which reveals a story of subtle dysregulation and high variability at the regulatory level. This suggests that the femtosecond laser does not just affect average expression levels, but it can disrupt the normal communication and decision-making network of the bacteria, leading to a loss of coordinated gene expression across the population. This heterogeneity could be a strategy for survival under stress, which agrees with Fig. 5a, because femtosecond laser treatment significantly increased the variability in QS gene expression (increased standard deviation in the treated groups T15 and T30 compared to the tight, consistent expression seen in the control group), suggesting network destabilization. The expression variability further increased, especially in *AgrA*, *ArlR*, and *SarA* genes. The dysregulation hypothesis might further explain the lack of translation of the transcriptional increase we saw with adhesion genes *clfB* and *fnbA*. For bacterial proteins to be functional, they need to be expressed in a coordinated manner^65^. The increased variability in QS regulators suggests the coordinating circuitry of the cell is disrupted^66^. The lack of concordance between transcriptional signals and phenotypic outcomes is a known consequence of severe cellular stress and regulatory network disruption^67^. Confirmation that the femtosecond laser-induced oxidative stress could cripple the cell’s ability to maintain the precise timing and stoichiometry would require measurement of functional protein expression^68^.

To further explore the overall correlation between gene expression changes, we performed correlation matrix analysis. Pearson R values for each group were calculated and plotted to produce correlation heatmaps, one for the control group in Fig. 5b and another for the T15 group in Fig. 5c. Given the statistical limitations we faced with only 3 data points, we aimed to use correlation analysis to look for broad, consistent patterns that could tell a biological story. The control group showed very strong, seemingly organized patterns, with blocks of very high positive correlation (red) and strong negative correlation (blue). On the other hand, there was a dramatic alteration of virulence gene co-expression networks after T15 treatment, even within systems (*Sae* and *Arl* System), indicating a wholescale disruption of the regulatory circuitry that governs virulence factor expression. Further validation with a larger sample size is required to confirm these findings.

While the box plots in Fig. 4 helped to illustrate the variability and statistical significance of the raw ΔCq values, the lollipop plot of the LFC shown in Fig. 6 offers a clear consolidated view of the overall treatment effect in all the genes. The LFC metric normalizes the data to the control, allowing for an immediate and quantitative assessment of the magnitude and direction of the changes, furthermore, it allows for the clear ranking of all investigated genes, which helps to pinpoint the primary transcriptional drivers of the observed phenotypic changes, such as the dramatic upregulation of adhesion genes *clfB* (LFC = 5.44, ~ 42-fold increase) and *fnbA* (LFC = 2.88, ~ 7.3-fold increase), which could be a misguided response attempting to compensate for the femtosecond laser-induced stress. However, this compensatory mechanism is foiled by the femtosecond laser’s double-edged sword effect on the *ica* operon. Our data shows a concurrent and significant downregulation of *icaA* (LFC = -0.658, ~ 1.58-fold decrease) while *icaD* (LFC = 1.975, ~ 3.9-fold increase) was upregulated. This decoupling within the same operon is a direct sign of severe transcriptional disruption, as suggested by the increased variability in QS regulators (Fig. 5a). A deficiency in *icaA* expression would probably lead to the production of incomplete or non-functional PIA^69^, thereby compromising effective cell-cell adhesion and consequently reducing biofilm formation. This explains the biofilm biomass reduction observed in Fig. 2b, further confirming that the effect of 400 nm femtosecond laser not only compromises bacterial viability but also impairs the bacteria’s ability to maintain and synthesize new EPS components despite the initial pro-adhesion signals, a result strongly confirmed by the SEM images and the reduction of the EPS matrix fraction (%) in Fig. 3.

**Fig. 11:**
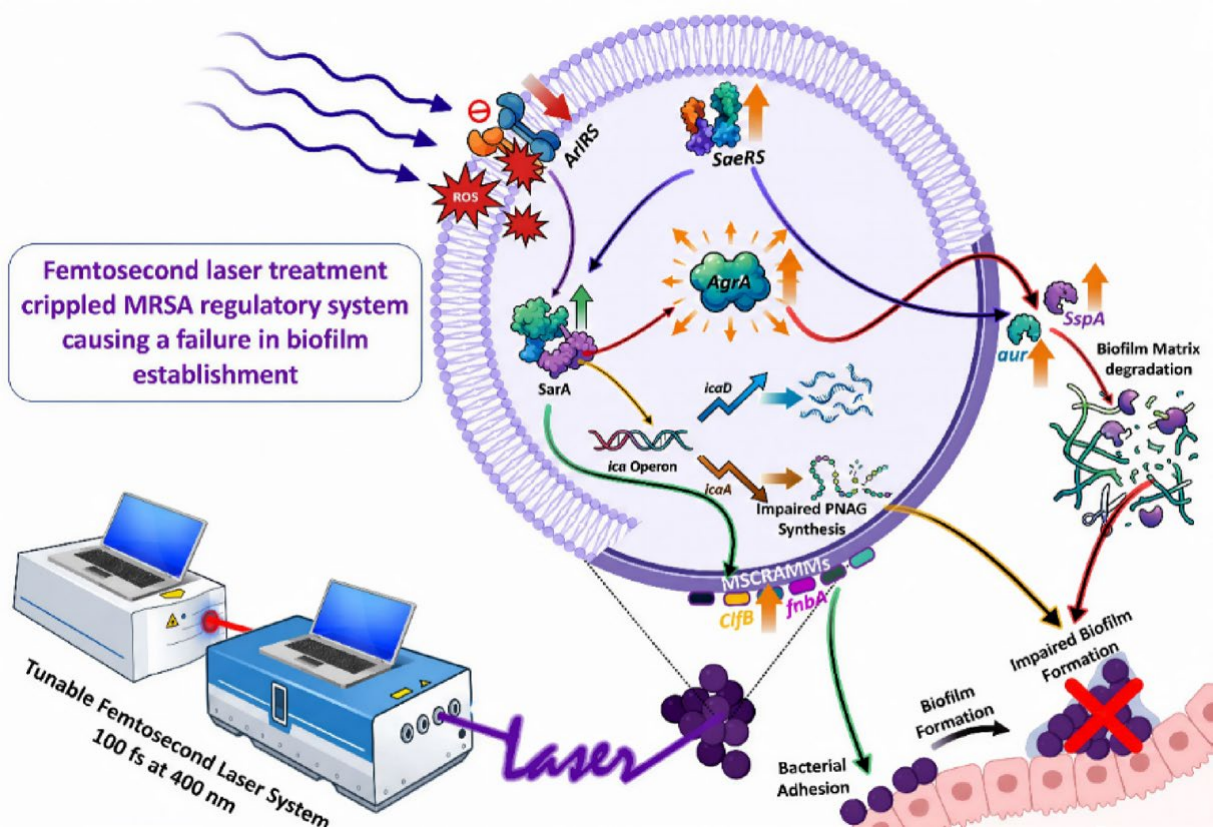
A diagram illustrates the proposed regulatory cascade by which 400 nm femtosecond laser treatment crippled MRSA quorum-sensing activity and decoupled the *ica* operon, causing a failure in biofilm establishment. Femtosecond laser-induced oxidative stress downregulated the *ArlRS* system, which allowed the *SarA* to surge, coordinating multiple downstream pathways simultaneously. Upregulation of the *SaeRS* system reinforced the high-stress state of the cells. The *agr* system was activated, unleashing proteases (*SspA* and *aur*) that degrade the biofilm matrix. A fantastic observation was the decoupling within the *ica* operon, with the downregulation of *icaA* impairing PNAG polysaccharide synthesis, weakening the biofilm’s physical structure. MRSA cells attempted to produce new adhesins (*clfB* and *fnbA*), but matrix degradation signals overwhelmed the attachment efforts, fundamentally weakening the biofilm structure.

To elucidate the mechanisms underlying the observed transcriptional changes shown in Fig. 7, we investigated the predicted protein-protein interaction (PPI) network between various regulatory systems and virulence factors. Within this network, key global regulators such as *SarA* and *SaeS* have emerged as central hubs, confirming their established role as master regulators of virulence in *S. aureus*. *SarA* was significantly upregulated (LFC = 1.608, ~ 3-fold increase) and is known to positively regulate the *Agr* quorum-sensing system^70^. *AgrA* showed a slight upregulation (LFC = 0.142, ~ 1.1-fold increase). Even a modest increase in *Agr* activity could signal a shift towards a planktonic lifestyle and cause biofilm dispersal by upregulating extracellular proteases^71^. This direct link could explain the observed upregulation of biofilm dispersion genes, *SspA* (LFC = 0.408, ~ 1.3-fold increase) and *Aur* (LFC = 0.708, ~ 1.6-fold increase). Thus, the initial attachment mediated by *clfB* (LFC = 5.44, ~ 42-fold increase) and *fnbA* (LFC = 2.88, ~ 7.3-fold increase) might be compromised by this concurrent activation of dispersal mechanisms. *SarA* is a known positive regulator of the *ica* operon transcription^72,73^; nevertheless, the observed downregulation of *icaA* (LFC = -0.658 ~ 1.58-fold decrease) suggests a novel stress response. The *SaeRS* system often interacts with other regulatory systems such as *SarA* and *Agr*, which are often activated by host defenses (e.g., neutrophils, ROS). The slight upregulation of the two *SaeRS* components; the histidine kinase *SaeS* (LFC = 0.275, ~ 1.2-fold increase) and the response regulator *SaeR* (LFC = 1.108, ~ 2.2-fold increase), may contribute to the increased expression of virulence genes, such as *fnbA*, *aur*, and *SspA*^74^. On the other hand, *ArlS* (LFC = -1.458, ~ 2.75-fold decrease) is a membrane-bound histidine kinase, a key sensor for cell stress such as the oxidative stress induced by the femtosecond laser, which could inhibit autophosphorylation and phosphotransfer to *ArlR* (LFC = -0.692 ~ 1,6-fold decrease). The *ArlRS* two-component system is known to be a repressor of *SarA* under certain conditions, so its downregulation means it cannot repress it, but it could also contribute to the *clfB* and *fnbA* upregulation^75^. The high degree of connectivity within the network provides a clear explanation for the diverse and widespread transcriptional changes observed in the virulence factors and other regulators as illustrated in the following Fig. 11. While our molecular analysis focused on the 15 min (T15) interval, the data from the CFU, crystal violet and MTT assays demonstrate that the maximum disruption of early biofilm formation occurs at the 45 min (T45) mark. This suggests a dose-dependent transition from an initial transcriptional dysregulation to a more profound and irreversible regulatory disruption. Even though the observed dysregulation of the quorum-sensing network provides a compelling model of network-wide shifts, protein-level validation and testing against a wider range of clinical MRSA isolates will be essential to confirm the clinical generalizability of these findings.

Given the known resilience of well-established biofilms, the destruction of a pre-existing, mature biofilm is a much harder challenge than the prevention of its formation. Nevertheless, our results clearly demonstrate the significant, although less pronounced, destructive capability of the femtosecond laser against 24-hour-old MRSA biofilms (Fig. 8). The efficacy of 400 nm femtosecond laser irradiation in eradicating established 24-hour MRSA biofilms was definitively quantified using the CFU assay. As illustrated in Fig. 8a, b, the treatment induced a significant and dose-dependent reduction in the number of viable cells within the mature biofilm architecture. The potent 69% reduction in viable MRSA cells within mature biofilms at T45 suggests that the 400 nm femtosecond laser treatment is exceptionally effective at penetrating the established biofilm architecture. To complement the viability data, the effect of femtosecond laser treatment on the physical architecture of the biofilm was quantified in Fig. 8c, d. The treatment induced a clear, dose-dependent reduction in biofilm biomass. When varying the average power at a fixed exposure duration (15 min), a significant reduction was achieved only at the highest power of 150 mW (P150), whereas lower powers (P50 and P100) showed negligible effects on the total biomass. In contrast, the exposure duration exerted a far more pronounced impact on biofilm integrity. Increasing the irradiation time at a constant power of 50 mW resulted in a significant biomass reduction reaching a maximum of 27.7% at 45 min (T45; d = 4.5, *p* < 0.0001). These findings indicate that the total delivered energy, particularly when extended over longer durations, is a critical factor in the gradual physical disruption of the biofilm matrix. This high penetrative capacity is likely due to the generated ROS, particularly hydroxyl radicals and singlet oxygen, which are known to induce the oxidative cleavage of polysaccharide chains and the cross-linking or fragmentation of matrix proteins^76,77,78^.

The physiological state of the surviving bacterial population, as measured by the MTT assay in Fig. 8e, f, reveals a corresponding decline in metabolic activity. The efficacy of the treatment was strongly dependent on exposure duration, where the 15-minute exposure (T15) reduced viability by 27.0%, which increased progressively to a 55.7% reduction in viable cells after a 45-minute exposure (T45) (d = 8.6, *p* < 0.001). This progressive increase in destruction suggests a cumulative effect, where the anti-biofilm effect is continuously active. The non-linear progression from T15 to T45 may indicate an initial phase of matrix disruption followed by accelerated bacterial cell death once the protective architecture has been compromised. In contrast, the assessment of variations in femtosecond laser power revealed a more complex and intriguing pattern. We observed a non-linear, inverse relationship between laser power and destructive effects, where the lowest power setting (P50) was surprisingly the most effective, causing a 29.6% reduction in viability (d = 4.3, *p* < 0.001). Conversely, the highest power setting (P150) was the least effective, with only a 17.2% reduction. This inverse relationship suggests a shift in the mechanism of action. At lower power (P50), the primary mechanism is likely to be photochemical (ROS production), which is highly effective at disrupting the cell’s internal machinery. At higher powers (P100 and P150), the effect may shift towards a more photothermal mechanism (heating).

The morphological analysis of 400 nm femtosecond laser irradiation efficacy in disrupting established 24-hour MRSA biofilms is visually and quantitatively confirmed by high-resolution SEM analysis in Fig. 9. The SEM micrographs at 10,000x and 20,000x magnification reveal a dramatic shift from the healthy, densely packed, uniform *S. aureus* cocci in the untreated control to a highly fragmented and damaged architecture in the treated groups. The first signs of cytotoxicity were membrane blebs and pits, suggesting the initial phase of membrane perforation at 15 min of exposure. The damage progressed to a severe degree after 30 min of treatment, where the biofilm architecture was largely disassembled, with individual cells showing extreme shrinkage, complete collapse (ghost cells), and widespread rupture. The presence of extensive extracellular debris and the creation of void spaces between the lysed cells are clear indicators of massive cytoplasmic leakage and cell death. At the most effective power setting (P50), the damage appeared more widespread and uniform across the cells. While the high-power treatments (P100 and P150) revealed intense, localized zones of fused or “melted” material, characteristic of heat-induced damage. This localized damage that the higher powers caused to the outermost layer of the biofilm could paradoxically shield the deeper layers from the femtosecond laser, reducing its overall penetration and efficacy. These findings strongly suggest that a longer exposure duration at a lower power intensity could be the optimal therapeutic parameters for destroying mature MRSA biofilms.

A notable observation in our quantitative SEM analysis of mature 24-hour biofilms is the relative stability of biofilm area coverage (%) across the treated groups. This finding is scientifically consistent with the nature of mature biofilm architecture. Since the femtosecond laser treatment was applied to an already established, multi-layered scaffold. Unlike the formation inhibition model, where the femtosecond laser prevents the initial assembly of the scaffold. The primary evidence of the femtosecond laser’s therapeutic success is found in the significant reduction of cell density (cells/µm²) and the dramatic increase in cell structural damage (%), which correlates with the reduced viability observed in the CFU assay. While our biofilm biomass results in Fig. 8c, d, indicated a significant reduction in total biomass (27.7% at T45), the matrix fraction (%) as quantified from SEM images showed more subtle changes. This highlights the methodological limitations of SEM-based image analysis in mature models. In a 24-hour biofilm, the EPS is already densely formed and heavily cross-linked. Even after the laser-induced oxidative cleavage of matrix components, the physical remnants of the matrix often persist on the surface. Furthermore, automated image analysis algorithms may struggle to differentiate between “intact” matrix and “damaged” matrix remnants that still occupy physical space.

In Fig. 10, the temporal response of MRSA biofilms to femtosecond laser irradiation was analyzed. Prolonged exposure ensures a continuous flux of ROS, therefore a cumulative photochemical effect that gradually degrades the structural integrity of the matrix and destroys the MRSA cells. The relatively high R^2^ values indicate that exposure duration could be a reliable and controllable parameter for achieving significant biofilm destruction. This allows for precise estimation of treatment durations required to achieve, for example, 50% biofilm metabolic inactivation, the minimum effective time, to be 40.5 min, 99% metabolic inactivation (1% viability) to be 79.3 min, and complete biofilm metabolic inactivation estimated at 99.9% to be 80.1 min, with a consistent inactivation rate of 1.26% per minute. which is invaluable for optimizing clinical protocols, minimizing collateral damage to host tissues, and ensuring effective eradication of persistent MRSA biofilms.

The application of femtosecond lasers in antibiofilm research has traditionally focused on surface modification. For example, Gnilitskyi et al.^79^ demonstrated that femtosecond laser-modified stainless-steel surfaces could reduce biofilm biomass by altering surface topography and amyloid production. Similarly, Cunha et al.^81^ and Luo et al.^80^ showed that laser-induced periodic surface structures (LIPSS) on titanium and other materials significantly reduce the initial adhesion of *S. aureus*^80,81^. Furthermore, Siddiquiea et al.^82^ explored sub-micron topographies on elastomeric surfaces to prevent biofouling^82^. However, these approaches are primarily preventative and rely on modifying the substrate before bacterial contact. In contrast, our study demonstrates that direct 400 nm femtosecond laser irradiation can effectively eradicate established 24-hour MRSA biofilms and prevent formation by directly targeting the bacteria’s internal regulatory mechanisms. This suggests that direct laser treatment could serve as a non-invasive clinical modality for treating localized biofilm-associated infections.

The therapeutic effectiveness of aPDT depends on the irradiation parameters of the light source, such as operational mode (continuous wave or pulsed), intensity, fluence, exposure duration, and, most critically, wavelength. These parameters have a major impact on the photon-tissue interactions, the target photosensitizer that absorbs the photon energy, the amount of energy delivered, and the rate of this transfer^83,84^. Recent progress in pulsed laser system technology, particularly femtosecond lasers, which can deliver energy rapidly to a precise area without affecting the surrounding tissue, is expected to revolutionize laser-based treatment modalities^85^. Femtosecond laser-based aPDT could have many advantages, such as low absorption by biological samples, more precise targeting, low phototoxicity, and clean, noninvasive, controllable laser pulses of nonionizing radiation^86,87^.

Overall, the rendered biofilm viability, metabolic inactivation, matrix disruption, and morphological quantification (SEM) data in this study provide a comprehensive, multi-dimensional understanding of the double-edged anti-biofilm efficacy of 400 nm femtosecond laser-based aPDT that is both statistically robust and clinically significant. On one hand, 400 nm femtosecond laser-based aPDT was able to prevent the formation of MRSA biofilms. While previous work has shown that sub-lethal aPDT can alter virulence gene expressions^88^, our research takes a critical step forward by trying to uncover the underlying regulatory mechanism. This finding is particularly significant as it positions femtosecond laser-based aPDT as a sophisticated anti-virulence modality. On the other hand, we also confirmed that 400 nm femtosecond laser treatment is a potent modality for eradicating established MRSA biofilms, aligning with a growing body of literature^89,90^. The optimization of femtosecond laser-based aPDT protocols requires a delicate balance between sufficient energy delivery and the avoidance of counterproductive photothermal shielding. An optimized strategy involving longer exposure at lower power was most effective for destroying mature biofilms. These results provide a clear and essential roadmap for developing next-generation aPDT systems capable of overcoming the challenge of chronic, biofilm-related infections.

## Conclusion and future perspectives

The present study has opened a new and critical perspective on the effect of femtosecond laser-based aPDT in mitigating MRSA biofilm infections. We have comparatively evaluated the femtosecond laser-based aPDT exposure parameters for optimum attenuation of bacterial biofilm formation and destruction. Our findings demonstrate that exposure to a femtosecond laser at 400 nm with an average power of 50 mW for an exposure duration of 15, 30, and 45 min effectively impaired MRSA biofilm formation ability, with 75.3% reduction in viability, 44.5% biomass reduction, and a 60–85% reduction in metabolic activity achieved at 45 min. The comprehensive gene analysis revealed a mechanism far more sophisticated than just simple bacterial killing. 400 nm femtosecond laser treatment appeared to create an environment where the bacterial response paradoxically prioritizes dispersal or a planktonic state, which led to a functional failure in biofilm establishment, despite the transcriptional attempt of the bacteria to compensate. Furthermore, our study provides novel insights into the destruction of established biofilms. We demonstrated that the anti-biofilm effect was strongly dependent on the exposure duration, with 69% reduction in viability, 27.7% biomass reduction, and a 55.7% reduction in metabolic activity achieved at 45 min. Crucially, the comparison of time and power-dependent destruction revealed a therapeutic window effect, where a lower power setting (P50) was more effective than higher powers (P100, P150). This response could be mechanistically explained by the shift from a broad, effective photochemical mechanism at low power to a more localized, self-shielding photothermal mechanism at high power. In conclusion, this work highlights the necessity for optimization of femtosecond laser parameters for both anti-virulence and biofilm destruction. It could be anticipated that future clinical implementation would lead to a promising antibacterial modality against multidrug-resistant bacterial infections.

## Supplementary Information

Below is the link to the electronic supplementary material.

Supplementary Material 1

## Data Availability

The data analysed during the current study are available from the corresponding author on reasonable request.
